# Clinical diagnostic values of transfer RNA-derived fragment tRF-19-3L7L73JD and its effects on the growth of gastric cancer cells

**DOI:** 10.7150/jca.51567

**Published:** 2021-04-02

**Authors:** Yijing Shen, Yaoyao Xie, Xiuchong Yu, Shuangshuang Zhang, Qiuyan Wen, Guoliang Ye, Junming Guo

**Affiliations:** 1 Department of Gastroenterology, The Affiliated Hospital of Medical School, Ningbo University, Ningbo 315020, China.; 2 Department of Biochemistry and Molecular Biology, and Zhejiang Key Laboratory of Pathophysiology, School of Medicine, Ningbo University, Ningbo 315211, China.; 3 Ningbo No. 1 Hospital Affiliated to Ningbo University School of Medicine.; 4 Institute of Digestive Diseases of Ningbo University, Ningbo 315020, China.

**Keywords:** gastric cancer, tRF-19-3L7L73JD, biomarker, proliferation, migration, apoptosis, cell cycle.

## Abstract

**Background and aim:** Medicine has made great progress, but gastric cancer is still one of the most common malignant tumors worldwide. tRNA-derived fragments (tRFs), a type of small non-coding RNA, have been found to play important roles in cancers. Due to an abundance of modifications, tRFs have the potential to serve as cancer biomarkers. However, the relationship between tRFs and gastric cancer is still largely unclear. We have identified a new tRF, tRF-19-3L7L73JD, found to be expressed at a lower level in gastric cancer patients than healthy controls. Our study aims to explore the diagnostic value of tRF-19-3L7L73JD screening in gastric cancer and to investigate its effects on the growth of gastric cancer cells.

**Methods:** Using quantitative reverse transcription-polymerase chain reaction, we identified tRF-3L7L73JD as differentially expressed in plasma from gastric cancer patients compared to healthy controls. We measured tRF-3L7L73JD levels in plasma from 40 gastric cancer patients and healthy controls. Furthermore, we tested another cohort containing 89 gastric cancer patients and 98 healthy controls to validate our findings. Next, we analyzed the relationship between levels of tRF-19-3L7L73JD in plasma and clinicopathological data of gastric cancer patients, and then evaluated the effects of tRF-19-3L7L73JD on gastric cancer cell growth. Cell proliferation was measured by the Cell Counting Kit‐8 and clone formation experiments after transfer with tRF-19-3L7L73JD mimics. The changes in cell migration ability were explored through the scratch and Transwell experiments. Finally, we explored changes in apoptosis and cell cycle by flow cytometry.

**Results**: tRF-19-3L7L73JD showed lower expression in the tested gastric cancer patients. In the validation cohort tRF-19-3L7L73JD was also expressed at low levels in the pre-operative plasma group compared with healthy plasma and post-operative plasma groups. Additionally, a comparison of gastric cancer cell lines with normal gastric epithelial cell lines produced the same result. We found that tRF-19-3L7L73JD expression in patients was related to tumor size. The area under the curve (AUC) was 0.6230, with sensitivity and specificity of 0.4045 and 0.7959, respectively. Cellular function studies revealed that tRF-19-3L7L73JD inhibited cell proliferation and migration, induced apoptosis, and arrested cells at G_0_/G_1_ phases, suggesting it may suppress the development of gastric cancer.

**Conclusion**: The results suggest that tRF-19-3L7L73JD may be useful as a biomarker of gastric cancer.

## 1. Introduction

Cancers are considered one of the most important factors affecting public health worldwide. Gastric cancer, a common malignancy, has the second-highest mortality rate among all malignancies in the world, with approximately 650,000 yearly deaths 1. Due to a lack of screening methods, it is difficult to diagnose the early stage of gastric cancer. There are no clinical data to support that existing tumor biomarkers, including carbohydrate antigen 125 (CA125), CA19-9, and carcinoembryonic antigen (CEA), are effective for the diagnosis of early gastric cancer 2. Most patients with gastric cancer have already reached middle or late stages when diagnosed 3. Early diagnosis and treatment are the most effective ways to reduce gastric cancer mortality. Therefore, it is crucial that new and efficient biomarkers for gastric cancer are identified.

tRNA-derived small RNAs (tsRNAs), one of the small non-coding RNAs (sncRNAs), are generated from matured tRNAs or pre-tRNAs and have various functions. Researchers have discovered tsRNAs exist in different species from *E. coli* to mammals 4. tsRNAs are not products of random degradation but are produced in specific cells or tissues under special conditions. tsRNAs can be roughly divided into two categories: tRNA halves (tiRNAs) and tRNA-derived fragments (tRFs). According to the cleavage site located in the anticodon, tiRNAs can be further divided into 5'tiRNAs and 3'tiRNAs. tRFs can be roughly divided into tRF-5s, tRF-3s, tRF-2s, tRF-1s, and i-tRFs 4,5.

Recently, multiple studies have found that tsRNAs play a key role in cell proliferation 6, 7, DNA damage response 8, translation regulation 9, gene silencing, and various human diseases 10. Researchers have found that tRFs regulate gene expression by combining with Argonaute (Ago) proteins and PiWi proteins 6. tRFs derived from tRNA^Glu^, tRNA^Asp^, and tRNA^Tyr^ were shown to affect the stability of oncogenic transcripts by competitive binding with Y box binding protein 1 mRNA 11. Recent studies have explored the possibility of tRFs as new biomarkers of diseases 12. Here, we focused on tRF-19-3L7L73JD, which is produced by the degradation of the precursor tRNA^Val-AAC^. The reasons for choosing tRF-19-3L7L73JD as the research target are mainly as follows: Our previous RNA sequencing results showed that tRF-19-3L7L73JD was one of the significantly differentially expressed tRFs between gastric cancer patients and healthy controls 7. This result was supported by quantitative reverse transcription-polymerase chain reaction (qRT-PCR) of the test group. Based on these findings, we further explored tRF-19-3L7L73JD expression in another cohort (validation group) and studied its effects on gastric cancer cells. The results showed tRF-19-3L7L73JD to have a tumor suppressive effect on gastric cancer.

## 2. Materials and methods

### 2.1. Plasma samples and clinical data collection

The clinical plasma samples used in this study were collected from two departments of gastroenterology: the Ningbo No. 1 Hospital Affiliated to Ningbo University School of Medicine and The Affiliated People's Hospital, Ningbo University, between February 2011 and June 2018. The test group included 40 plasma samples from gastric cancer patients and 40 plasma samples from healthy controls. The validation group included 89 matched pairs of plasma samples of gastric cancer patients 1 day before and 7 days after surgery, and plasma samples from 98 healthy people. The criteria for the inclusion of patients enrolled into this study were: greater than 18 years of age, confirmation of disease by histopathology, and no receipt of any treatments (radiotherapy, chemotherapy, biological targeted therapy, or combination therapy). The exclusion criteria for patients were: concurrent pregnancy or breast-feeding, diagnosis of other cancers or serious diseases, or prior receipt of treatments (radiotherapy, chemotherapy, biological targeted therapy, or combination therapy).

All plasma samples were centrifuged immediately after collection and the supernatant was stored in a -80 °C freezer before use. Clinical staging of tumors was classified according to the rules of the International Union Against Cancer Tumor Lymph Node Metastasis (TNM) Staging System Eighth Edition 13. Written informed consent was obtained from all subjects. The Human Research Ethics Committee of Ningbo University approved all aspects of this study.

### 2.2. Total RNA extraction and its quality determination

Total RNA from samples was extracted by TRIzol LS (Invitrogen, Carlsbad, CA, USA) following the manufacturer's instructions and then measured using a SmartSpec Plus Spectrophotometer (Denovix, Hercules, CA, USA). The purity of total RNA was evaluated according to the A260/A280 ratio. A ratio between 1.8-2.0 was considered to be acceptable for the experiment. Total RNA was stored at -80 °C until needed.

### 2.3. RNA pretreatment

RNA was pretreated with the rtStarTM tRF and tiRNA Pretreatment Kit (Arraystar, Rockville, MD, USA) according to manufacturer instructions to remove additional modifications (such as, demethylation, diacylation, or addition of terminal enzymes) so as not to interfere with subsequent qRT-PCR.

### 2.4. Reverse transcription

Pre-processed RNAs underwent reverse transcription using the rtStar ™ First-Strand cDNA Synthesis Kit (3' and 5' adaptors) (Arraystar) according to manufacturer instructions.

### 2.5. Quantitative reverse transcription-polymerase chain reaction

To detect the expression level of tRF-19-3L7L73JD, qRT-PCR was performed using GoTaqqPCR Master Mix (Promega, Madison, WI, USA) on a Mx3005P real-time PCR system (Stratagene, Palo Alto, CA, USA). U6 small nuclear RNA was used as reference. All Primers (Supplemental Table 1) were synthesized by Sangon Biotech (Shanghai, China). Amplification of tRF-19-3L7L73JD was confirmed by sequencing (Supplemental Figure 1). The expression level of tRF-19-3L7L73JD was determined using the 2^-ΔΔ*C*t^ method for relative quantification 14,15. All experiments were independently repeated three times.

### 2.6. Cell culture

The human gastric epithelial cell line GES-1 and gastric cancer cell lines SGC-7901, AGS, and BGC-823 were purchased from the Shanghai Institute of Biochemistry and Cell Biology, Chinese Academy of Sciences. AGS and BGC-823 were derived from primary non-metastatic tumors and were less differentiated, and SGC-7901 originated from lymphatic metastatic tumors and was moderately differentiated. GES-1, SGC-7901, and BGC-823 cell lines were cultured using Roswell Park Memorial Institute (RPMI) 1640 medium (HyClone, Los Angeles, CA, USA), supplemented with 1% penicillin/streptomycin (Life Technologies, Carlsbad, CA, USA) and 10% fetal bovine serum (Gibco, Grand Island, NY, USA). AGS cell lines were cultured in Dulbecco's modified Eagle's medium (DMEM) high glucose (HyClone), containing 1% penicillin/streptomycin and 10% fetal bovine serum. All cell lines were cultured at 37°C and 5% CO_2_ atmosphere.

### 2.7. Transfection

Fully confluent cells (3×10^5^ cells per well) were first seeded into 6-well plates (Corning, New York, NY, USA) and then incubated in complete medium for approximately 24 h until growth reached 60%-70% confluence. SGC-7901 and BGC-823 cells were transiently transfected by adding 5 μl negative control (NC) or tRF-19-3L7L73JD mimics (GenePharma, Shanghai, China) and 5 μl Invitrogen™ Lipofectamine 2000 (Life Technologies) per 200 μl Opti-MEM I Reduced Serum Medium (Invitrogen, Carlsbad, CA, USA). Follow-up experiments were performed after cells 24 h-48 h after transfection.

### 2.8. Cell proliferation analysis

Cell proliferation was measured by Cell Counting Kit‐8 (CCK-8) assay (Dojindo, Tokyo, Japan) and cell clone counting assay. For CCK-8, cells were seeded into a 96-well plate (Corning) at a density of 5000 cells per well 24 h after transfection. After culture for 24 h, 48 h, 72 h, and 96 h, 10 μl of CCK-8 reagent was added to each well and incubated for 3 h. The SpectraMax M5 multifunctional microplate reader (Molecular Devices, Silicon Valley, CA, USA) was used to analyze optical density (OD) at 450 nm. Every experiment was repeated with five wells. For the cell colony counting, 24 h after transfection, cells were seeded in a 6-well plate with 1000 cells per well, changing to complete medium after 7 days. Cells were stained with crystal violet (Solarbio, Beijing, China) after 14 days and images of the cells were counted with Photoshop software (Adobe, San Jose, CA, USA). Each experiment was independently repeated three times.

### 2.9. Cell migration analysis

The cell scratch test and Transwell migration assay were used to verify whether migration ability was changed after upregulation of the tRF-19-3L7L73JD mimic. The cell scratch test was observed at 0 h and 48 h under the CKCG3 microscope (Olympus, Tokyo, Japan) 24 h after transfection. For the Transwell experiment, cells were grown for 24 h after transfection and then digested with 0.25% trypsin- ethylenediaminetetraacetic acid (EDTA). After centrifugation, cells were washed with phosphate buffer saline (PBS) and resuspended in serum-free medium. Then, cells were transferred at a concentration of 80,000 cells per well to a 24-well plate. In the lower chamber, 500 μl complete medium containing 15% fetal bovine serum was added, while 200 μl cell suspension in the same medium was added to the upper chamber. After incubation for 24 h, cells were fixed with 4% paraformaldehyde and stained with crystal violet. Finally, cells were observed and counted under an inverted microscope. All experiments were independently repeated three times.

### 2.10. Cell apoptosis analysis

Cell apoptosis was assessed by flow cytometry (BD FACSCalibur, Franklin Lake District, NJ, USA) after propidium iodide (PI) staining. According to the manufacturer's instructions, the transfected cells were digested with EDTA-free trypsin and washed twice with PBS. Then cells were resuspended in 500 μl Binding Buffer (Multisciences, HangZhou, China) at room temperature. Next, 10 μl PI and 5 μl Annexin V-FITC (Multisciences) were added and the cells were placed on ice for 5 mins in a dark environment. Once completed, cell apoptosis was analyzed by flow cytometry. All experiments were independently repeated three times.

### 2.11. Cell cycle analysis

The transfected and starved cells were harvested and washed twice with PBS. For cell staining, 1 mL DNA staining solution and 10 µL IntraPrep permeabilization reagent were added to the cells and then incubated at room temperature for 30 min in a dark environment. Next, cell cycle distributions were analyzed by flow cytometry. All experiments were independently repeated three times.

### 2.12. Statistical analysis

Statistical analyses were performed using SPSS V.19.0 software (IBM, Almont, NY, USA). GraphPad prism 5 software (GraphPad Software Inc, San Diego, CA, USA) and Image J software (Rawak Software Inc, Stuttgart, Germany) were also used. Comparison of tRF-19-3L7L73JD expression was analyzed by *t*-tests between plasma samples from before and after the operation in patients with gastric cancer and healthy controls. Changes in cell proliferation, cell migration, cell apoptosis, and cell cycle caused by up-regulation of tRF-19-3L7L73JD were also analyzed by using *t*-tests. The χ^2^ test was used to analyze the relationship between tRF-19-3L7L73JD expression level and clinicopathological factors of patients with gastric cancer. *P* < 0.5 was considered to be statistically significant.

## 3. Results

### 3.1. Differences of tRF-19-3L7L73JD levels in plasma of patients with gastric cancer and healthy controls

**Figure 1:**
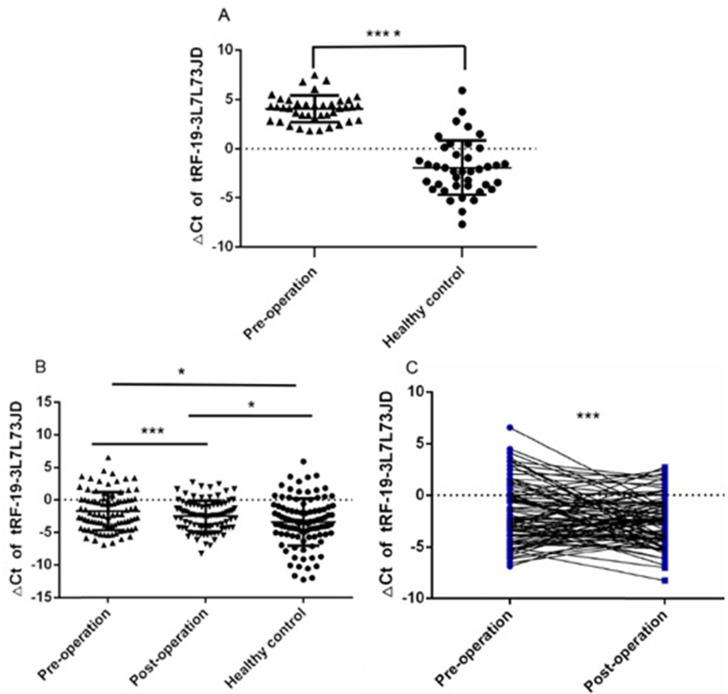
** The levels of tRF-19-3L7L73JD in plasma from patients with gastric cancer and healthy controls.** A) Tested group: The levels of tRF-19-3L7L73JD in gastric cancer patients and healthy control, *n*=40, ^****^P < 0.0001. B) Validation group: The levels of tRF-19-3L7L73JD in pre-operation (*n*=89), post-operation (*n*=89), and healthy subjects (*n*=98). ^*^P < 0.05, ^***^P < 0.001. C) the levels of tRF-19-3L7L73JD in paired pre-operative and post-operative samples. *n*=89, ^***^P < 0.001.

We first detected the expression of tRF-19-3L7L73JD in the test group of plasma samples from 40 gastric cancer patients and healthy controls by qRT-PCR. Figure 1A shows that there are differences in tRF-19-3L7L73JD expression between the patients and controls. Next, we detected the expression of tRF-19-3L7L73JD in the validation group including 89 pairs of plasma samples from gastric cancer patients 1 day before and 7 days after surgery, and 98 healthy controls. The results indicate that, compared to healthy controls, the levels of tRF-19-3L7L73JD in both pre-operative and post-operative groups are lower (Figure 1B, 1C).

### 3.2. The relationship between levels of tRF-19-3L7L73JD in plasma and clinicopathological factors, and its potential diagnostic value

**Figure 2:**
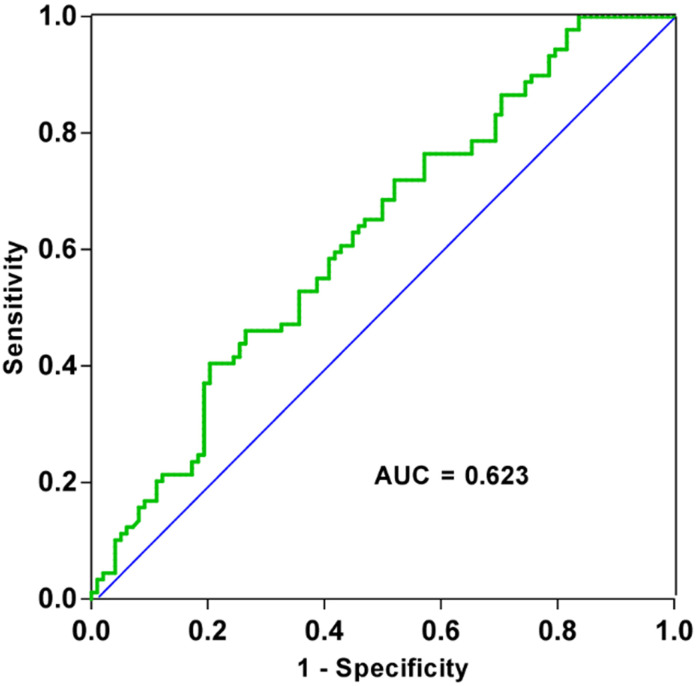
ROC curve of tRF-19-3L7L73JD.

After analyzing patient clinicopathological data, we observed that the levels of tRF-19-3L7L73JD in plasma were related to some characteristics. First, we divided patients into high-expression and low-expression groups based on qRT-PCR results and whether the ∆*C*_t_ value for each sample was more or less than average. We then analyzed the relationship between levels of tRF-19-3L7L73JD and pathological factors. Table 1 shows that levels of tRF-19-3L7L73JD are related to tumor size. As shown in Figure 2, the area under the receiver operating characteristic (ROC) curve of tRF-19-3L7L73JD is 0.6230. The sensitivity and specificity are 0.4045 and 0.7959, respectively. These results suggest the potential value of tRF-19-3L7L73JD in the screening of gastric cancer.

### 3.3. Expression levels of tRF-19-3L7L73JD in gastric cancer cell lines

**Figure 3:**
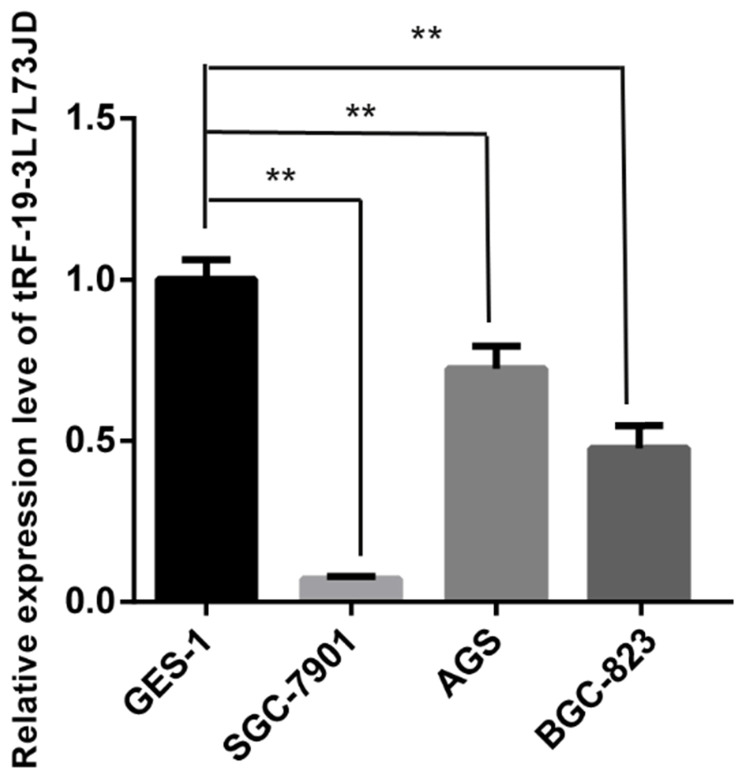
Expression levels of tRF-19-3L7L73JD in normal gastric epithelial cell line GES-1 and gastric cancer cell lines, SGC-7901, AGS, and BGC-823. Relative expression was calculated using the 2^-∆∆*C*t^ method.^ **^P < 0.01. All results are expressed as means ± SD of three independent experiments.

After detection of tRF-19-3L7L73JD levels in plasma (Figure 1), we further examined expression level differences of tRF-19-3L7L73JD between the normal gastric epithelial cell line GES-1 and three gastric cancer cell lines, SGC-7901, AGS, and BGC-823. Our results showed that compared with GES-1, the expression level of tRF-19-3L7L73JD in the three gastric cancer cell lines was lower (Figure 3), consistent with the expression trend of tRF-19-3L7L73JD observed in plasma samples.

### 3.4. tRF-19-3L7L73JD effect on gastric cancer cell proliferation, migration, apoptosis, and cell cycle

**Figure 4:**
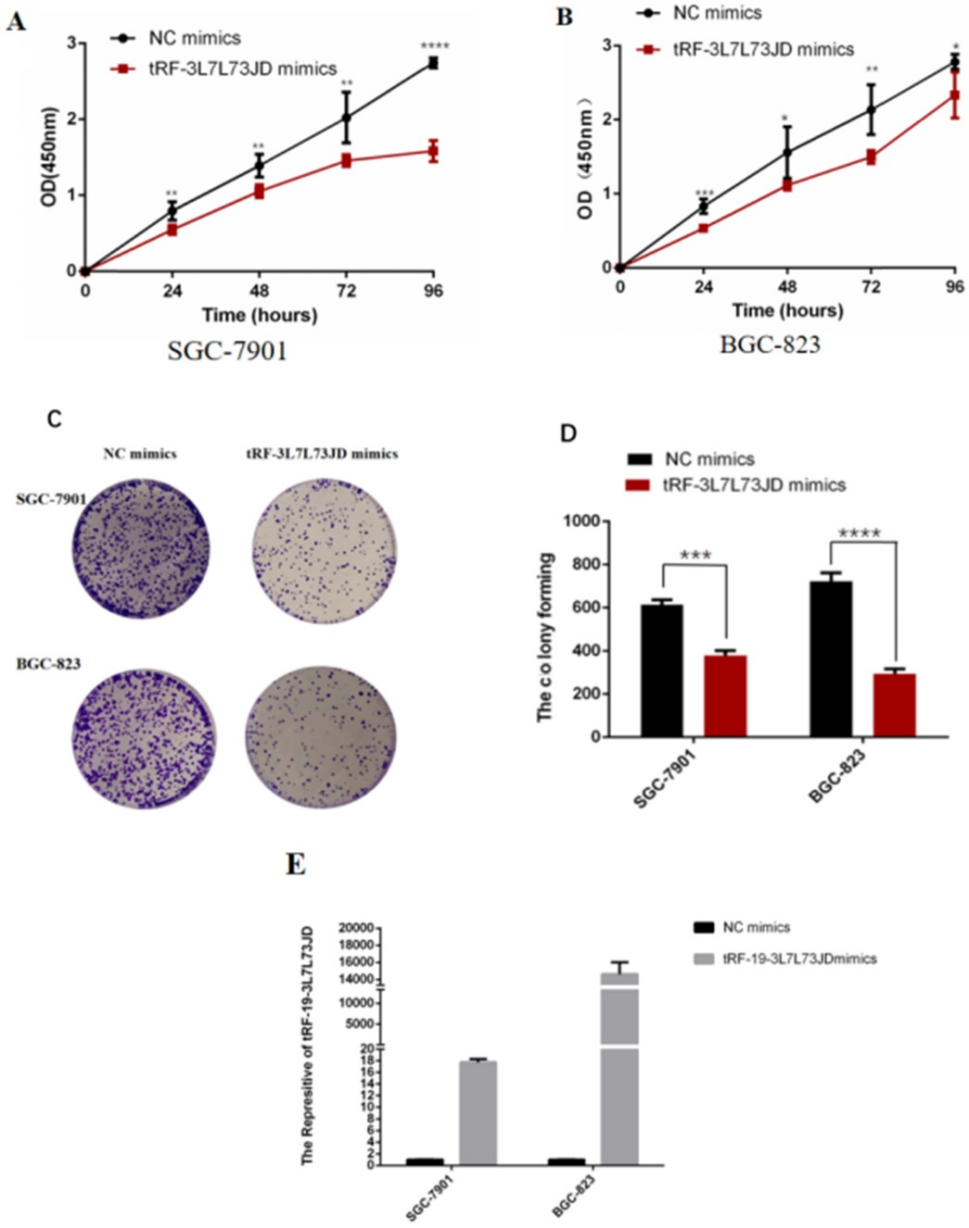
** Proliferation of gastric cancer cells after tRF-19-3L7L73JD up-regulation.** A) Growth curves of SGC-7901. B) Growth curves of BGC-823. C) Representative cell colony forming assay of SGC-7901 and BGC-823. D) A statistical chart of cell colonies. E) Effect of tRF-19-3L7L73JD up-regulation in gastric cancer cell lines. NC, negative control. *n*=3, ^*^P < 0.05, ^**^P < 0.01, ^***^P < 0.001, ^****^P < 0.0001.

We evaluated the effect of tRF-19-3L7L73JD up-regulation on cell proliferation. The CCK-8 results showed that compared with the negative control (NC), cell proliferation decreased gradually after up-regulation of tRF-19-3L7L73JD (Figure 4A and B). Cell colony forming assay also showed that the number of cells in the treated group was smaller than that in the NC group (Figure 4C and D).

**Figure 5:**
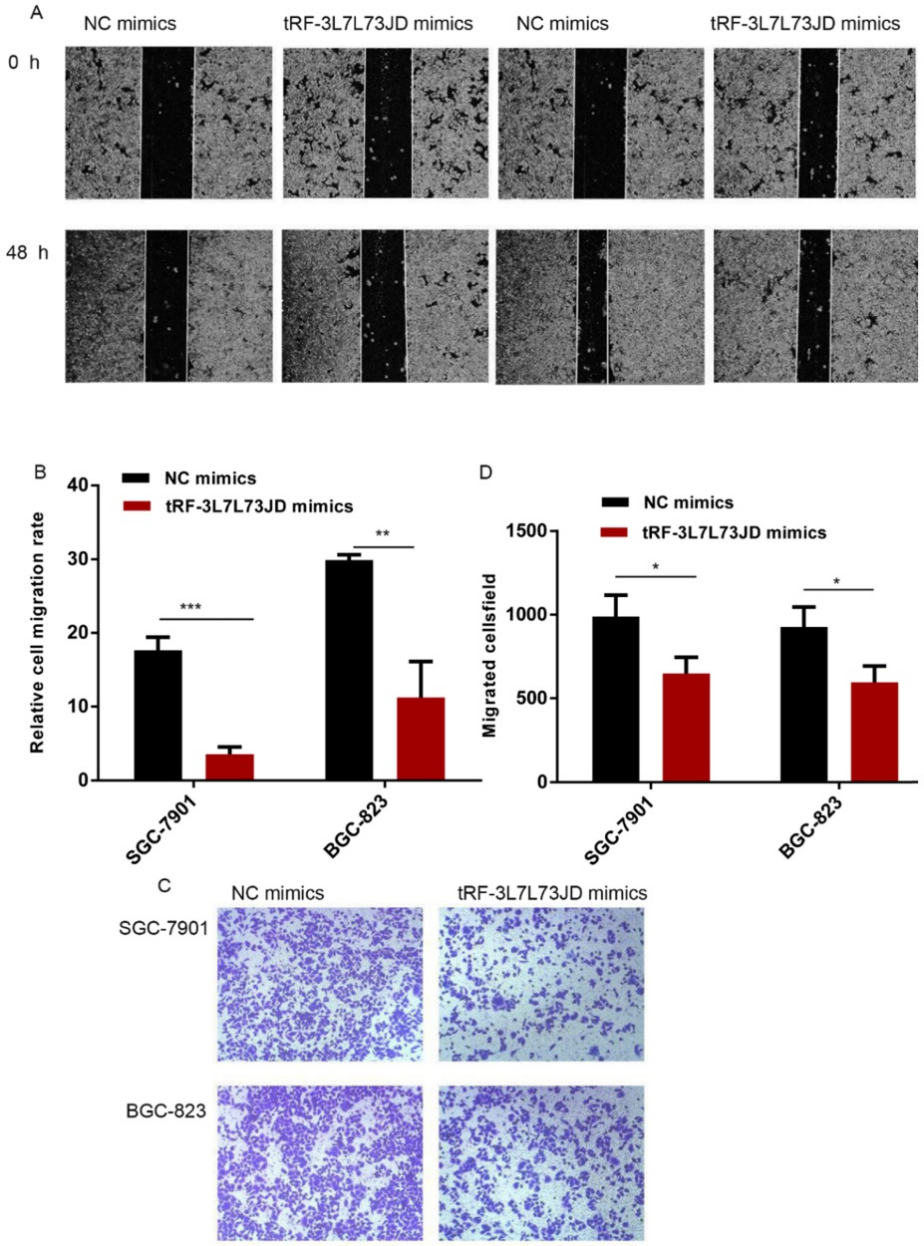
** Gastric cancer cell migration ability after up-regulation of tRF-19-3L7L73JD.** A) Representative results of scratch experiments. B) A statistical chart of scratch experiments. C) Representative results of the Transwell assay. D) A statistical chart of the Transwell assay. NC, negative control. *n*=3, ^*^P < 0.05, ^**^ *P* < 0.01, ^***^P < 0.001.

To detect changes in the migration ability of gastric cancer cells, we used the scratch test and Transwell assay. Compared with the NC group, the scratch experiment revealed that the migration capacity of gastric cancer cells was reduced after the expression level of tRF-19-3L7L73JD was increased (Figure 5A and B). Transwell experiments also showed similar results (Figure 5 C and D).

**Figure 6:**
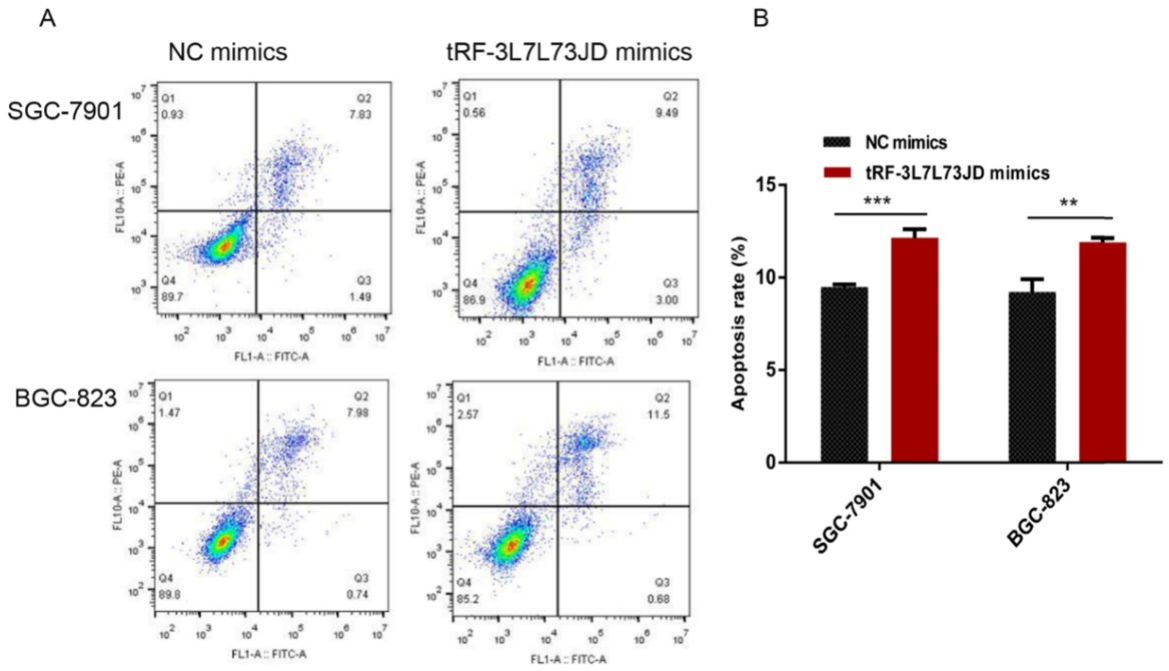
** Apoptosis changes in gastric cancer cells after tRF-19-3L7L73JD up-regulation.** A) Representative results. B) Statistical chart. NC, negative control. *n*=3, ^**^P < 0.01, ^***^P < 0.001.

**Figure 7:**
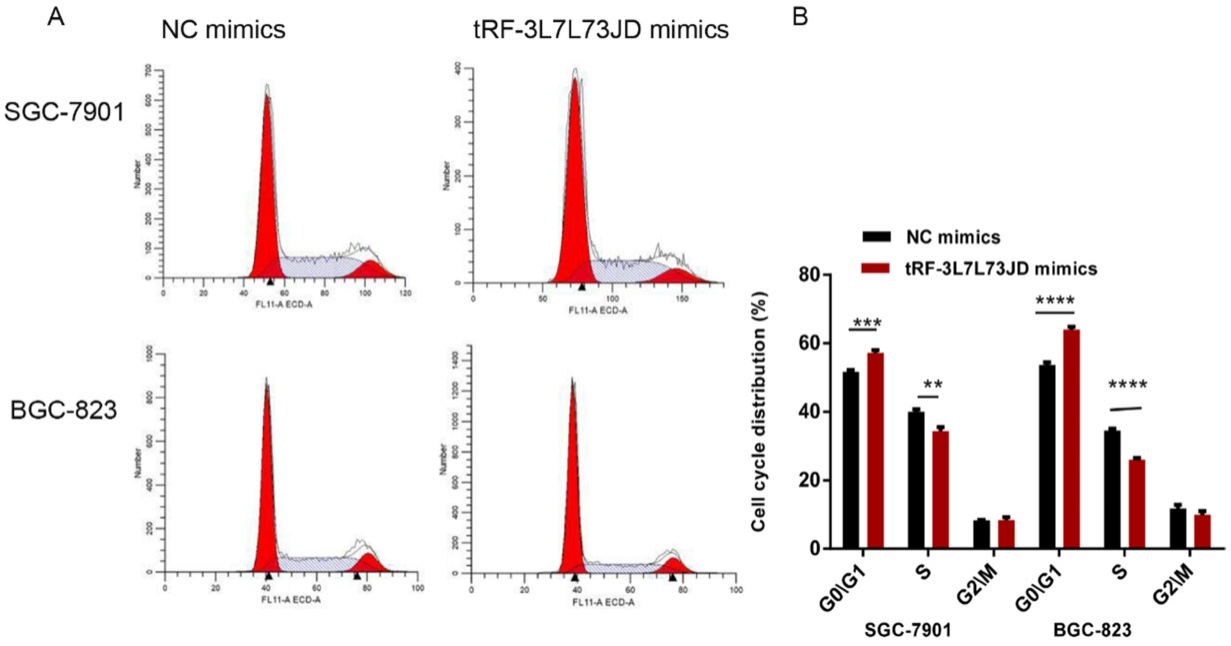
** Cell cycle changes of gastric cancer cells after tRF-19-3L7L73JD up-regulation.** A) Representative results. B) Statistical chart. NC, negative control. *n*=3, ^**^P<0.01, ^***^P < 0.001, ^****^P < 0.0001.

Our examination of cell apoptosis showed that the apoptosis rate of gastric cancer cell lines increased after transfection with tRF-19-3L7L73JD mimics (Figure 6). Cell cycle analysis showed that gastric cancer SGC-7901 and BGC-823 cells were arrested at G_0_/G_1_ phase after tRF-19-3L7L73JD up-regulation (Figure 7).

## 4. Discussion

Over the last decade, the maturity and widespread application of RNA sequencing technology has led to an increasing discovery of tsRNAs 16, 17, 18. However, the class of small tRNA-degrading molecules was once considered to be miRNAs 19. Eventually, researchers confirmed that tsRNAs were a unique type of sncRNAs with distinct biological functions 20, 21. The small degradation fragments of tRNAs were first discovered in a stress or starvation environment. However, investigators also found that tsRNAs are produced in cells without stress or starvation 22-24. Many sncRNA studies have begun to clarify their function 25, but studies of tsRNAs have been limited, and their functions remain undefined. Some researchers have found that tsRNAs can be produced by angiogenin (ANG) 23, 26. As a ribonuclease present in mammalian cells, ANG can respond to sudden stress. For example, to protect cells from apoptosis caused by osmotic pressure, ANG-cleaved tiRNAs competitively bind to cytochrome c 27. In addition, some tsRNAs have been found to have the same sequence as miRNAs and function in the same biological manner as miRNAs 21. The tsRNAs sequences derived from tRNA^Leu^, tRNA^Lys^, and tRNA^Ala^ are identical to the sequences of miR-1280, miR-1274a/b, and miR-886-5p, respectively 28. Haussecker et al. found that some tsRNAs control RNA silencing to regulate genes through differential AGO protein linkage 21. Yamasaki et al. found that translation was inhibited after 5'tiRNA was transfected into the human osteoblast cell line U2OS, due to the oligoguanine motif at its end 23.

tsRNAs have been identified as members of the gene regulatory network, which controls many key pathophysiological processes and pathogenic risk factors, such as neurodegenerative diseases, cancers, metabolic diseases, pathological stress damage, and so on 5, 10, 29. Although the mechanisms of most ncRNAs in regulating diseases are still unknown 30-32. There are now several studies that prove the existence of highly enriched tsRNAs in various cell types 33-36. For example, tsRNAs participate in various biological functions such as proliferation and migration of various tumor cells 37-40.

It has been recognized that tsRNAs may be promising new targets and biomarker candidates of some cancers. In our study, qRT-PCR results showed lower levels of tRF-19-3L7L73JD in plasma samples from patients with gastric cancer (Figure 1). The clinicopathological data indicated that the levels of tRF-19-3L7L73JD were related to tumor size (Table 1). Moreover, tRF-19-3L7L73JD showed its diagnostic value (Figure 2). Compared with normal gastric epithelial cells, the expression of tRF-19-3L7L73JD was lower in gastric cancer cell lines (Figure 3). As is known, tumor size is an important factor affecting patient prognosis. In addition, compared to other sncRNAs, tsRNAs are stable in plasma due to many modifications 41, 42. As a result, we considered tRF-19-3L7L73JD to be a potential biomarker of gastric cancer.

In subsequent experiments on the biological functions of tRF-19-3L7L73JD on gastric cancer cells, we found that proliferation, migration, apoptosis, and cell cycle changed after the expression level of tRF-19-3L7L73JD was increased in gastric cancer cells (Figure 4, 5, 6, and 7), providing us with evidence that tRF-19-3L7L73JD may play a tumor suppressor role in gastric cancer.

## 5. Conclusions

We found that up-regulation of tRF-19-3L7L73JD can inhibit the proliferation and migration of gastric cancer cells, promote apoptosis, and affect cell cycle. This study provides potential new therapeutic targets for gastric cancer.

## Supplementary Material

Supplementary figure and table.

## Figures and Tables

**Table 1:** The relationship of tRF-19-3L7L73JD levels (∆*C*_t_) in plasma with clinicopathological factors of patients with gastric cancer.

Factors	Samples (%)	Low (%)	High (%)	*P*
**Cases**	89 (100)	45 (50.6)	44 (49.4)	
**Gender**				
Male	60 (67.4)	31 (68.9)	29 (65.9)	0.7643
Female	29 (32.6)	14 (31.1)	15 (34.1)	
**Age (y)**				
≥60	69 (77.5)	34 (75.6)	35 (79.5)	0.6521
<60	20 (22.5)	11 (24.4)	9 (20.5)	
**CEA**				
Positive	14 (15.7)	8 (17.8)	6 (13.6)	0.5916
Negative	75 (84.3)	37 (82.2)	38 (86.4)	
**CA19-9**				
Positive	15 (16.9)	10 (22.2)	5 (11.4)	0.1713
Negative	74 (83.1)	35 (77.8)	39 (88.6)	
**Differentiation**				
Well	58 (65.2)	26 (57.8)	32 (72.7)	0.1389
Moderate-Poor	31 (34.8)	19 (42.2)	12 (27.3)	
**Tumor-size (cm)**				
≤5	65 (73.0)	27 (60.0)	38 (86.4)	0.0051
>5	24 (27.0)	18 (40.0)	6 (13.6)	
**TNM stage**				
0 & I	33 (37.1)	15 (33.3)	18 (40.9)	0.4028
II	18 (20.2)	10 (22.2)	8 (18.2)	
III	36 (40.4)	20 (44.4)	16 (36.4)	
IV	2 (2.2)	0 (0.0)	2 (4.5)	
**Invasion**				
Tis	3 (3.4)	2 (4.4)	1 (2.3)	0.6822
T1	25 (28.1)	12 (26.7)	13 (29.5)	
T2 & T3	11 (12.4)	4 (8.9)	7 (15.9)	
T4	50 (56.2)	27 (60.0)	23 (52.3)	
**Lymphatic metastasis**				
N0	39 (43.8)	20 (44.4)	19 (43.2)	0.0739
N1	22 (24.7)	7 (15.6)	15 (34.1)	
N2 & N3	28 (31.5)	18 (40.0)	10 (22.7)	
**Distal metastasis**				
M0	87 (97.8)	45 (100.0)	42 (95.5)	0.1480
M1	2 (2.2)	0 (0.0)	2 (4.5)	
