# Supplementary material for: Clinical diagnostic values of transfer RNA-derived fragment tRF-19-3L7L73JD and its effects on the growth of gastric cancer cells
Source: J Cancer. 2021 Apr 2;12(11):3230–8. doi: 10.7150/jca.51567 (PMC8100793; doi:10.7150/jca.51567)
Supplement: Supplementary file 1 — Supplementary figure and table. [file jcav12p3230s1.pdf]

Supplemental data

Supplemental Table 1. qRT-PCR Primer sequences

**Name**	**Sequence**
tRF-19-3L7L73JD	F: 5' CCGACGATCCCGTAGTGTAG3'
	R: 5' TGCTCTTCCGATCTGATAACC3'
U6	F: 5' GCTTCGGCAGCACATATACTAAAAT 3'
	R: 5’ CGCTTCACGAATTTGCGTGTCAT 3’

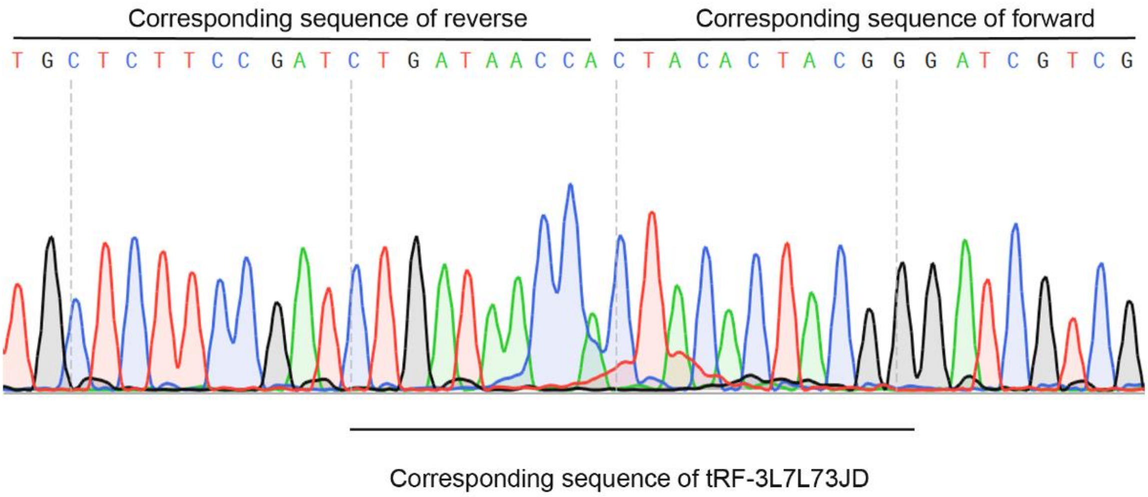

Supplemental Figure 1. Sequencing results of tRF-19-3L7L73JD qRT-PCR products.
